# Vaccine Hesitancy and Fear of COVID-19 Among Italian Medical Students: A Cross-Sectional Study

**DOI:** 10.1007/s10900-022-01074-8

**Published:** 2022-02-09

**Authors:** Giuseppina Lo Moro, Eleonora Cugudda, Fabrizio Bert, Immacolata Raco, Roberta Siliquini

**Affiliations:** Department of Public Health Sciences, University of Turin, Via Santena 5 bis, 10126 Turin, Italy

**Keywords:** COVID-19, Vaccine hesitancy, Medical students, Fear of COVID-19

## Abstract

Medical students are in close contact with patients and should adhere to the same recommendations as healthcare workers. The study aimed to explore medical students' hesitancy towards COVID-19 vaccine and evaluate fear of COVID-19 and its relationship with hesitancy. A cross-sectional survey was conducted amongst a sample of medical students attending clinical years (November 2020–February 2021, Italy). Multivariable regressions were performed (p < 0.05 as significant). A total of 929 students participated (58.6% of eligible students). Hesitancy was reported by 6.7%; extreme fear of COVID-19 by 42.0%. Among hesitancy predictors, there were the survey completion before COVID-19 vaccine authorisation (adjOR = 6.43), adverse reactions after a vaccination (adjOR = 3.30), and receiving advice against COVID-19 vaccination from a relative (adjOR = 2.40). Students who received the recommended paediatric vaccinations (adjOR = 0.10), students with higher adherence to preventive measures (adjOR = 0.98), and students with fear of contracting COVID-19 with regard to the health of loved ones (adjOR = 0.17) were less likely to be hesitant. Females (adjOR = 1.85), students with poor health (adjOR = 1.64), students who had a loved one severely affected by COVID-19 (adjOR = 1.68), and students with fear of contracting flu (adjOR = 3.06) had a higher likelihood of reporting extreme fear. Hesitancy was remarkably lower than in similar studies. However, there is room for improvement in university activities that could deepen the competence in vaccines. Our focus on fear should not be overlooked, since the extent of extreme fear that we found might represent a substantial burden, considering the associations between fear and other health outcomes.

**Supplementary Information:**

The online version contains supplementary material available at 10.1007/s10900-022-01074-8.

## Introduction

Vaccine confidence is central to the success of a vaccination campaign [1]. 'Vaccine Hesitancy' has been defined as ‘delay in acceptance or refusal of vaccination despite the availability of vaccination services' [2] and, in 2019, the World Health Organization (WHO) identified vaccine hesitancy as a major threat to global health [3]. Thus, vaccine hesitancy must be considered when planning COVID-19 vaccination campaigns.

A systematic review on acceptance of COVID-19 vaccination (when available) across 33 countries (as of 25th December 2020) reported that Kuwait (23.6%), Jordan (28.4%), and Italy (53.7%) had the lowest rate [4]. In addition, in earlier Italian studies, the prevalence of COVID-19 vaccine hesitancy was 29.5% and 41% [5, 6].

In particular, special attention should be paid to medical students: they are in close contact with patients and should adhere to the same recommendations as healthcare workers (HCWs) [7]. Several studies have explored medical students' knowledge and attitudes towards vaccinations [8, 9]. The most important predictor of vaccination confidence was having been previously vaccinated, so achieving a high vaccination coverage among students today might mean having a high coverage among HCWs in the future [10–12]. Interestingly, the European Medical Students' Association found that, although almost all medical students and young doctors recognised the need for booster vaccination schedules, only 68.0% got the recommended ones [13]. The current unprecedented context and the recent extraordinary development and approval of COVID-19 vaccines [14] require investigations to question medical students’ attitudes towards COVID-19 vaccines, as they may be highly exposed to COVID-19 during hospital practices [15]. In the 2020 first semester, an Italian study on university students reported 13.9% were COVID-19 vaccine hesitant, with no differences between students in healthcare courses and those in other courses [16]. Remarkably, in December 2020, in a Polish study on university students, the majority of students desired to be vaccinated, with a higher percentage among medical students (92%) than among other students (59.4%) [17]. Nonetheless, studies focusing on medical students after the vaccine authorisation [14] are lacking.

Last, the COVID-19 pandemic has induced the development of fear, worries, and anxiety among people worldwide [18]. Medical students might represent a particular population as they can be at risk for poor mental health outcomes [19, 20] and the impact of the pandemic on their psychological health should not be overlooked, also in the light of potential relationships between fear and behaviours, such as vaccine hesitancy.

Therefore, the primary aim of this study was to assess medical students' COVID-19 vaccine hesitancy, along with attitudes and doubts towards vaccinations, analysing possible associations between vaccine hesitancy and students’ characteristics and experiences. The secondary aim was to assess the impact the pandemic had on medical students from a psychological point of view, exploring the fear of COVID-19 and whether this had repercussions on vaccination propensity.

## Methods

Between 20 November 2020 and 2 February 2021, a cross-sectional survey was conducted amongst students attending medical school at the University of Torino. The inclusion criteria were being a student currently enrolled in the clinical years (4th, 5th, and 6th year and students beyond the allotted time to attain the degree) and being able to understand the questionnaire in Italian. At the University of Torino, medical students begin clinical rotations starting from the 4th year and, since they are in close contact with patients, vaccinations (e.g. flu vaccinations) are encouraged and offered free of charge [21].

The research was conducted using the Computer-Assisted Web Interview (CAWI) method. Participants were recruited by convenience through emails. A total of 1584 students were eligible and received an email with the survey. Raosoft® was used to determine that the minimum sample size was 310, based on a 5% margin of error, 95% confidence level, 50% response distribution, and population of 1584 eligible students. People entering the survey received an explanation and an informed consent form. Participation was voluntary and anonymous, and participants received no compensation.

### The Questionnaire

The questionnaire had three sections.

The first section contained mainly socio-demographic items. Moreover, participants were asked if their financial situation had worsened due to the pandemic, if they had a family member who is a HCW, and if they or a loved one belonged to a vulnerable group at high risk for COVID-19 severe consequences.

The second section investigated attitudes towards vaccinations. Participants were asked whether they had received all the recommended paediatric vaccinations and whether those vaccinations caused any adverse effects. Attitudes and behaviours towards flu vaccination and COVID-19 vaccination were further explored. Students were asked if they had received the flu vaccination in the last three years, if they had ever received advice not to get vaccinated against flu or COVID-19, and if they had seen on social media the recommendation not to receive these vaccinations. Fear of contracting flu or COVID-19 was examined both with regard to the participant’s personal health and with regard to the health of loved ones with whom the participant is in contact. Students were asked if they were willing to get vaccinated against COVID-19 when the vaccine was available (answers “Yes”, “No”, “I do not know”). If students answered “No” or “I do not know”, they were labelled as “vaccine hesitant”. Vaccine hesitant students were asked if they would receive the vaccine after one year from the vaccine approval. Then, the grade of agreement with statements on vaccinations in general, the grade of agreement with statements on COVID-19, and the grade of agreement with statements on COVID-19 vaccinations were explored (5-item Likert-type answers from “strongly disagree” to “strongly agree”).

The third section examined COVID-19-related aspects. For instance, participants were asked if they had ever been isolated because of COVID-19, if they or a loved one tested positive for COVID-19, and if they or a loved one had health consequences because of COVID-19. Students were asked if they had ever exhorted relatives/friends to follow preventive measures. Attitudes and behaviours towards several preventive measures (during August 2020 and during October 2020) were investigated. In the region where the study was conducted, in August there were no restrictions and in October measures were gradually implemented up to a new lockdown in November 2020. Both regarding August and October, a percentage score to estimate the adherence to preventive measures was calculated (“preventive score”, description is in the Supplemental file: M1 and Table S3), where 100% represents the highest adherence.

Last, the Fear of COVID-19 Scale (FCS-19S) [22] was administered using the Italian validated version [23]. The FCS-19S is a tool for evaluating personal fear of COVID-19: it consists of 7 items (5-item Likert-type answers from “strongly disagree” to “strongly agree”). Summing the items, the score ranges from 7 to 35. The higher is the score, the greater is the fear. In the study sample, the Cronbach’s alpha of the FCV-19S was 0.839. A score of 16.5 or higher has been used as the cut-off for extreme fear, which correlates with symptoms of anxiety, anxiety for health, and post-traumatic stress disorder [24–26]. An extreme fear of COVID-19 represented the secondary outcome of the present study.

Additionally, the dates of completion of the survey were dichotomized in “before 21st December 2020” and “21st December 2020 and after”. On this date, the European Medicines Agency (EMA) authorised the first COVID-19 vaccine in Europe [14]. Moreover, after this approval, it was declared that medical students at the University of Torino would be vaccinated during an early phase of the campaign, right after HCWs.

### Statistical Analysis

Descriptive analyses were performed. Continuous variables did not have a normal distribution (Shapiro Wilk test) and were expressed as median and interquartile range (IQR). Chi-squared tests (Mann Whitney U tests where appropriate) were conducted to assess differences between hesitant and non-hesitant students and between students with and without an extreme fear.

To explore characteristics associated with the outcomes, univariable and multivariable logistic regressions were executed. The multivariable models, adjusted for age and gender, were achieved with a stepwise forward selection process, with a univariable p-value < 0.250 as the main criterion [27]. SPSS (v27) was used and a two-tailed p-value < 0.05 was considered statistically significant. Missing values were excluded.

## Results

A total of 929 students began the questionnaire (58.6% of eligible students) and, among them, 902 completed the questionnaire including at least the primary outcome. Therefore, 27 questionnaires were excluded from all analyses. The excluded participants were not different from the included participants for gender (p = 0.463), age (p = 0.506) or year of course (p = 0.959).

Female accounted for 63.5% and the median age was 24 years (IQR = 23–26). Participants that attended the 4th year, the 5th year, the 6th year, and participants outside prescribed time were 22.2%, 22.6%, 28.8%, and 26.4%, respectively. The overwhelming majority of participants were Italian (98.4%).

Concerning the primary outcome, 60 participants (6.7%) reported vaccine hesitancy (4 students declared “No” and 56 “I don’t know”). Among them, 44 students (73.3%) would receive the vaccine after 1 year from the vaccine approval, while 2 students (3.3%) still would not receive it and 14 (23.3%) were still unsure. About the secondary outcome, extreme fear was reported by 354 participants (42.0%). The median FCV-19S score was 15 (IQR = 12–19). The association between vaccine hesitancy and extreme fear was not significant (p = 0.238): 42.5% of non-hesitant students and 34.0% of hesitant students showed an extreme fear.

According to chi-squared tests, participants who completed the survey before 21st December, female students, and students into a relationship had significantly higher percentages of vaccine hesitancy. Extreme fear was significantly more frequent among females, non-smokers, students with a very poor/poor/fair health status or belonging to a vulnerable group for COVID-19, and participants with a loved one belonging to a vulnerable group for COVID-19. (Supplemental file: Table S1).

A total of 97.7% were aware of having received all the recommended paediatric vaccinations, 54.2% of participants did not receive flu vaccination in the 3 years before the survey, and 9.4% reported to have suffered from an adverse reaction after a vaccination. Participants who received advice not to get vaccinated against flu were 32.6% and participants who received advice not to get vaccinated against COVID-19 were 52.8%.

A total of 9.4% and 50.5% reported a moderate/high fear of personally contracting flu and COVID-19 (respectively) with regard to the participant’s own health. A total of 68.8% and 96.9% reported a moderate/high fear of personally contracting flu and COVID-19 (respectively) with regard to the health of loved ones. Regarding the pandemic, 87.7% had exhorted relatives/friends to follow preventive measures. The preventive score had a median of 77.8% (IQR 66.7–88.9%) in October and of 66.7% (IQR 55.6–77.8%) in August. A total of 12.5% of participants had a loved one tested positive for COVID-19 and severely affected by the disease.

The prevalence of vaccine hesitancy was significantly higher among participants who were not aware of having received all the recommended paediatric vaccinations, participants who suffered from an adverse reaction after a vaccination, participants who received advice from a relative not to get vaccinated against COVID-19, and participants who had never exhorted relatives/friends to follow preventive measures. The prevalence of extreme fear was significantly higher among students who had exhorted relatives/friends to follow preventive measures, students who had a loved one that was severely affected by COVID-19, and students who had a moderate/high fear of contracting flu and COVID-19 (concerning both their own health and the loved ones’ health). The distribution of the preventive measure score in August was the same across the categories of fear of COVID-19 (p = 0.069) and vaccine hesitancy (p = 0.150). The distribution of the preventive measure score in October was the same across the categories of fear of COVID-19 (p = 0.111), while it was different (p = 0.010) between hesitant people (median 66.7%, IQR 63.9–77.8%) and non-hesitant people (median 77.8%, IQR 66.7–88.9%). (Supplemental file: Table S2).

Considering the statements about vaccinations in general, the highest percentage of agreement for statements against vaccinations was reported for “I do not think to be adequately informed about vaccinations” (8.0%) and “I am afraid of needles” (4.0%). Considering the statements about COVID-19, the highest percentage of agreement was for “I do not think that my health is at risk in case of infection” (18.1%), “I would not want to be among the first individuals to get vaccinated” (8%), “Getting vaccinated is complicated for logistical reasons” (7.2%). Considering the statements about COVID-19 vaccination, the highest percentage of disagreement for pro-vaccination statements was reported for “Vaccination should be mandatory” (11.3%), “It is essential that I get vaccinated to protect myself” (4.3%), and “I do not think we will return to normal life until most people are vaccinated” (4.0%).

Vaccine hesitancy had a significant association with most of the statements: overall, it was associated with agreement with anti-vaccination statements and with disagreement with pro-vaccination statements. Extreme fear of COVID-19 did not have any significant associations with any statement about general vaccinations. About COVID-19, extreme fear was significantly more frequent among students that agreed with “It is essential that I get vaccinated to protect myself”, “I am afraid I am allergic to some component of the vaccine”, and “Vaccination should be mandatory”. Last, extreme fear was significantly more frequent among students that disagreed with “I do not think that my health is at risk in case of infection” and “I would not want to be among the first individuals to get vaccinated”. (Supplemental file: Table S4).

The multivariable model of the first outcome (Table 1) showed that participants who completed the survey before 21st December, participants who experienced adverse reactions after a vaccination, and participants who were advised from a relative not to get vaccinated against COVID-19 had a higher likelihood of reporting vaccine hesitancy. Students who were aware of having received all the recommended paediatric vaccinations and students with fear of contracting COVID-19 with regard to the health of loved ones had a lower probability of being hesitant. The higher was the preventive score in October, the lowest was the likelihood of vaccine hesitancy. (Univariable regressions are shown in the Supplemental file: Table S5).

**Table 1:** Multivariable logistic regression model for vaccine hesitancy

	N	Vaccine hesitancy	
		adjOR (95% CI)	p-value
Age	838	1.11 (0.99–1.25)	0.076
Gender			
Male	312	Ref	
Female	526	1.64 (0.79–3.42)	0.183
Living alone			
No	786	Ref	
Yes	52	0.15 (0.02–1.32)	0.087
Worsening of economic status due to the pandemic			
No	698	Ref	
Yes	140	2.06 (0.98–4.33)	0.058
Having a family member who is a HCW			
No	569	Ref	
Yes	269	1.93 (0.99–3.77)	0.053
Survey before 21st December			
No	617	Ref	
Yes	221	6.43 (3.26–12.67)	** < 0.001**
Being aware of having received all the recommended paediatric vaccinations			
No	18	Ref	
Yes	820	0.10 (0.03–0.39)	**0.001**
Having suffered from an adverse reaction after a vaccination			
No	759	Ref	
Yes	79	3.30 (1.43–7.64)	**0.005**
Having seen on social media the recommendation not to receive the flu vaccination			
No	435	Ref	
Yes	403	0.51 (0.26–0.997)	**0.049**
Having ever received advice not to get the COVID-19 vaccination: from a relative			
No	632	Ref	
Yes	206	2.40 (1.21–4.77)	**0.012**
Fear of personally contracting COVID-19: with regard to the health of loved ones			
No	258	Ref	
Yes	580	0.17 (0.05–0.58)	**0.005**
Preventive score (October)	838	0.98 (0.96–0.99)	**0.013**

838 observations used in the model. Participants presenting the outcome “vaccine hesitancy” were 49 out of 838 (5.9%)

*adjOR* adjusted odds ratio, *CI* confidence interval, *HCW* healthcare worker, *N* sample size, *OR* odds ratio

The multivariable model of the second outcome (Table 2) revealed that females, participants with a very poor/poor/fair health status, and participants who had a loved one that was severely affected by COVID-19 had a higher likelihood of reporting extreme fear. Students with fear of contracting flu (both regarding the participant’s own health and the health of loved ones) were more likely to show extreme fear of COVID-19. (Univariable regressions are shown in the Supplemental file: Table S6).

**Table 2:** Multivariable logistic regression model for extreme fear of COVID-19

	N	Extreme fear	
		adjOR (95% CI)	p-value
Age	841	1.01 (0.96–1.07)	0.645
Gender			
Male	314	Ref	
Female	527	1.85 (1.36–2.51)	** < 0.001**
Living with preschool children			
No	813	Ref	
Yes	28	0.44 (0.18–1.08)	0.073
Smoking			
No	714	Ref	
Yes	127	0.68 (0.45–1.04)	0.075
Very poor/poor/fair health status			
No	689	Ref	
Yes	152	1.64 (1.12–2.39)	**0.011**
Fear of personally contracting flu: with regard to the participant’s own health			
No	762	Ref	
Yes	79	3.06 (1.78–5.24)	** < 0.001**
Fear of personally contracting flu: with regard to the health of loved ones			
No	260	Ref	
Yes	581	1.7 (1.22–2.37)	**0.002**
A loved one tested positive for COVID-19			
No, never	361	Ref	0.075
Yes (not severely affected)	375	1.03 (0.75–1.4)	0.869
Yes (severely affected)	105	1.68 (1.06–2.67)	**0.029**
Fear of personally contracting COVID-19: with regard to the health of loved ones			
No	26	Ref	
Yes	815	2.6 (0.85–7.96)	0.093

841 observations used in the model. Participants presenting the outcome “extreme fear of COVID-19” were 354 out of 841 (42.1%)

*adjOR* adjusted odds ratio, *CI* confidence interval, *N* sample size, *OR* odds ratio

## Discussion

The primary aim of the present study was to assess medical students' vaccine hesitancy towards COVID-19 vaccinations. The secondary aim was to assess the psychological impact of the pandemic, studying the fear of COVID-19 and its potential relationship with vaccine hesitancy.

Most of the sample presented a positive attitude towards the COVID-19 vaccination: only 6.7% showed vaccine hesitancy. The prevalence of vaccine hesitancy against the COVID-19 vaccine in our sample was lower than the prevalence of vaccine hesitancy reported among the Italian general population (10–46.3%) [4–6, 28], among HCWs (22–39%) [29, 30], and among medical students (8–46%) [17, 31]. In our sample, worrying about adverse reactions against the COVID-19 vaccine (23 out of 60 hesitant students, 38.3%) was associated with vaccine hesitancy. However, in the available literature represented a much more consistent element (33 out of 37 hesitant students, 89.2%) [31]. Fear of being allergic to some component of the vaccine was significantly more common among hesitant participants (4 out of 60 hesitant students, 6.7%) than among non-hesitant (16 out of 842 non-hesitant students, 1.9%), although it is an extremely rare occurrence [32]. This element, along with the fact that many hesitant students did not consider themselves an important factor in the disease spread and that they would not vaccinate if SARS-Cov-2 circulation decreased much, represents a defective knowledge on vaccines and epidemiology, raising awareness on potential themes that could be deepened in curricular or extracurricular activities. The analysis showed also that hesitant students did not want to be among the first individuals to be vaccinated. Most of them were not convinced that a safe and effective vaccine could be developed so quickly. It is therefore not surprising that hesitant students (17 out of 60 hesitant students, 28.3%) believed that the COVID-19 vaccination should not be mandatory, to a lesser extent than the available literature (18 out of 37 hesitant students, 48.6%) [31]. This evidence was shown also for the influenza vaccine, for which vaccinated HCWs agreed more frequently with mandatory vaccination than non-vaccinated HCWs [33].

According to regression models, students who completed the questionnaire before 21st December, the date on which EMA authorised the first COVID-19 vaccine [14], were more likely to be hesitant. It could be explained as there was still great uncertainty on whether safe and effective vaccines would have been available in the near term. In this atmosphere of uncertainty, it does not surprise that those who had experienced adverse reactions after past vaccinations were significantly represented among the hesitant students. Finally, the likelihood of vaccine hesitancy increased among those who had a relative who advised against COVID-19 vaccination. Although participants reported that they were more frequently advised from other people than from relatives, receiving advice from relatives was the only one with a significant association with vaccine hesitancy. Thus, there seems to be a greater influence of advice received from relatives than from other people or advice found online.

Being aware of having received all the recommended paediatric vaccinations was a negative predictor of vaccine hesitancy. This is consistent with studies that found that one of the most important determinants of vaccination propensity was having previously been vaccinated, thus underlining the importance of achieving a high coverage among medical students today to have a high coverage among future HCWs [10–12].

Interestingly, an association between extreme fear of COVID-19 and vaccine hesitancy was not found. Instead, a negative association between vaccine hesitancy and moderate/high fear of personally contracting COVID-19 with regard to possible consequences for loved ones was shown. This suggests that medical students may feel the burden of being a potential carrier of disease rather than being infected. Indeed, fear of transmitting the disease to relatives has already been reported to be a positive predictor of vaccination readiness [17].

Finally, students who adhered to a higher number of preventive behaviours in October were less likely to be hesitant. This result is in line with other literature, which showed that the percentage of pro-vaccine individuals was higher among participants who agreed that preventive behaviour was an act of social responsibility [6].

Regarding the secondary outcome, 42.0% reported extreme fear of COVID-19. In our sample, the median FCV-19S score was 15 (IQR = 12–19), which is lower than in other studies, possibly due to lower mortality from COVID-19 within the study population (i.e. young people) and, therefore, a lower perceived risk. Indeed, in studies that considered the general population, the FCV-19S median scores ranged from 16 to above 20 [22, 34, 35]. This could be explained by the higher mean age of the population (so a higher mortality from COVID-19), by the period in which data were collected (until August 2020), and by the different epidemiology in the various geographical areas [22, 34, 35]. The FCV-19S median scores among university students and post-graduates who participated in international studies [36, 37] ranged from 16 to 19 points; this difference could be partially due to the different periods in which data were collected (until May 2020) and the different epidemiology of the areas (Pakistan and Spain, respectively) [36, 37]. In our sample, women were more likely to report extreme fear of COVID-19. During the pandemic and lockdown periods, women appeared to carry a greater burden than men [38] and were more likely to worry about the impact of COVID-19 [39, 40]. Also, students reporting a poor health status seemed to have an extreme fear, in line with other studies that found an association between fear and being part of an at risk group [34, 35]. Participants who reported a moderate/high fear of contracting flu, either in relation to consequences for their own health or for the loved one’s health, were more likely to show an extreme fear, thus suggesting they might have a different risk perception towards other infectious diseases too. The reasons behind this augmented risk perception should be further investigated. Understandably, students who had a loved one who was severely affected by COVID-19 had an extreme fear, consistently with other studies that found a significant association between fear and having had a family member died from COVID-19 [34].

This study had some limitations that should be acknowledged. The main limitations included the cross-sectional design, the opportunistic sampling, and the fact that no data about students who did not participate were collected. Another limitation was that the data collection was closed before the 7 April 2021, i.e. the date on which EMA confirmed the association between some dangerous thrombotic events and COVID-19 vaccines [41]. As our sample has a median age of 24 years, participants belonged to the age group with the most unfavourable risk/benefit ratio for vaccines such as Vaxzevria and Janssen [42]. Although medical students at the University of Torino did not receive Vaxzevria nor Janssen, it would be interesting to repeat the survey to assess how the events of the following months may have influenced their attitudes and beliefs.

Despite some limitations, this study is relevant since it involved a population whose opinion is of great interest in the wider context of vaccine hesitancy. Among the study’s strengths, there is the high response rate and the fact that it was one of the first Italian studies to quantify and evaluate the determinants of COVID-19 vaccine hesitancy among medical students. To our knowledge, this was the first Italian study to use the validated FCV-19S scale to investigate fear of COVID-19 and its determinants among medical students.

In conclusion, vaccine hesitancy among medical students in our sample proved to be remarkably lower than similar studies. However, there is still important room for improvement in terms of university activities that could deepen the competence in vaccines and epidemiology since students (especially if hesitant) showed wrong beliefs on these matters. Additionally, our secondary focus on extreme fear of COVID-19 should not be overlooked. Although the FCV-19S median scores were lower than in other studies, 42% of students reported an extreme fear of COVID-19: a substantial burden considering that associations between fear and other mental health outcomes exist [24] and that medical students are at risk for poor mental outcomes [19]. Moreover, the moderate/high fear detected against COVID-19 seems to go together with fear against flu, probably in a wider context of augmented perceived risk against infectious diseases that should be further investigated.

## Supplementary Information

Below is the link to the electronic supplementary material.

Supplementary file1 (DOCX 89 kb)

## Data Availability

All relevant data are within the paper. The datasets generated during and/or analysed during the current study are available from the corresponding author on reasonable request.
