# Supplementary material for: Vaccine Hesitancy and Fear of COVID-19 Among Italian Medical Students: A Cross-Sectional Study
Source: J Community Health. 2022 Feb 9;47(3):475–83. doi: 10.1007/s10900-022-01074-8 (PMC9160103; doi:10.1007/s10900-022-01074-8)
Supplement: Supplementary file 1 — Supplementary file1 (DOCX 89 kb) [file 10900_2022_1074_MOESM1_ESM.docx]

# Supplementary Tables

Table of contents

[Supplementary Tables 1](#_Toc83982253)

[Supplemental Methods 1](#_Toc83982254)

[M1. Preventive score development 1](#_Toc83982255)

[Supplemental Results 2](#_Toc83982256)

[Table S1. Characteristics of the sample: overall and stratified by the outcomes 2](#_Toc83982257)

[Table S2. Descriptive analysis of items about flu vaccination, COVID-19 and COVID-19 vaccination and their relationships with the outcomes 4](#_Toc83982258)

[Table S3. Descriptive analysis of the items of the preventive score 7](#_Toc83982259)

[Table S4. Statements about vaccinations in general and about COVID-19 and COVID-19 vaccinations and their relationships with vaccine hesitancy 9](#_Toc83982260)

[Table S5. Logistic regression models for vaccine hesitancy: univariable and multivariable regressions 12](#_Toc83982261)

[Table S6. Logistic regression models for extreme fear of COVID-19: univariable and multivariable regressions 13](#_Toc83982262)

## Supplemental Methods

### M1. Preventive score development

Attitudes and behaviours towards several preventive measures (in August and October 2020) were investigated. In Italy, in August there were no restrictions and in October measures were gradually implemented up to a new lockdown in the region where the study was conducted in November 2020. Both regarding August and October, a score to estimate the adherence to preventive measures was calculated (“preventive score”). One point was assigned if students: washed/sanitized frequently their hands; practised a proper respiratory etiquette; used an app for contact tracing; reduced the use of public transport; increased the cleaning of frequently touched surfaces; met relatives/friends with masks and distancing. One point was assigned if students did not: touch often their face; go to restaurants/bars or similar with people outside their household; go to indoor gatherings. After summing these points, a percentage score was calculated, where 100% represents the highest adherence.

## Supplemental Results

### Table S1. Characteristics of the sample: overall and stratified by the outcomes

| **Characteristic** |  | **Overall** | **Vaccine hesitancy** | | | **Extreme fear** | | |
| --- | --- | --- | --- | --- | --- | --- | --- | --- |
|  |  | **(n=902)** | **No**  **(n=842)** | **Yes**  **(n=60)** | **p** | **No (n=489)** | **Yes (n=354)** | **p** |
|  |  | N (%) | N (%) | N (%) |  | N (%) | N (%) |  |
| **Age*** |  | 24 (23-26) | 24 (23-26) | 24 (23-26) | 0.162 | 24 (23-25.5) | 24 (23-26) | 0.703 |
| **Date of survey completion** | *21^st^ Dec or after* | 660 (73.2) | 634 (96.1) | 26 (3.9) | **<0.001** | 359 (57.9) | 261 (42.1) | 0.919 |
|  | *Before 21^st^ Dec* | 242 (26.8) | 208 (86.0) | 34 (14.0) |  | 130 (58.3) | 93 (41.7) |  |
| **Gender** | *Male* | 328 (36.5) | 315 (96.0) | 13 (4.0) | **0.013** | 213 (67.8) | 101 (32.2) | **<0.001** |
|  | *Female* | 570 (63.5) | 523 (91.8) | 47 (8.2) |  | 274 (52.0) | 253 (48.0) |  |
| **Into a relationship** | *No* | 400 (44.3) | 382 (95.5) | 18 (4.5) | **0.021** | 226 (60.1) | 150 (39.9) | 0.268 |
|  | *Yes* | 502 (55.7) | 460 (91.6) | 42 (8.4) |  | 263 (56.3) | 204 (43.7) |  |
| **Living alone** | *No* | 870 (93.6) | 785 (93.0) | 59 (7.0) | 0.119 | 458 (58.0) | 331 (42.0) | 0.926 |
|  | *Yes* | 59 (6.4) | 57 (98.3) | 1 (1.7) |  | 31 (57.4) | 23 (42.6) |  |
| **Living with preschool children** | *No* | 872 (96.7) | 815 (93.5) | 57 (6.5) | 0.454 | 468 (57.4) | 347 (42.6) | 0.064 |
|  | *Yes* | 30 (3.3) | 27 (90.0) | 3 (10.0) |  | 21 (75.0) | 7 (25.0) |  |
| **Living with people aged 65 years or more** | *No* | 723 (80.2) | 673 (93.1) | 50 (6.9) | 0.523 | 395 (58.8) | 277 (41.2) | 0.368 |
|  | *Yes* | 179 (19.8) | 169 (94.4) | 10 (5.6) |  | 94 (55.0) | 77 (45.0) |  |
| **Economic status of family worsened due to the pandemic** | *No* | 747 (82.8) | 702 (94.0) | 45 (6.0) | 0.097 | 414 (59.1) | 287 (40.9) | 0.169 |
|  | *Yes* | 155 (17.2) | 140 (90.3) | 15 (9.7) |  | 75 (52.8) | 67 (47.2) |  |
| **Smoking** | *No* | 760 (84.3) | 710 (93.4) | 50 (6.6) | 0.839 | 404 (56.5) | 311 (43.5) | **0.037** |
|  | *Yes* | 142 (15.7) | 132 (93.0) | 10 (7.0) |  | 85 (66.4) | 43 (33.6) |  |
| **Health status** | *Good/*  *Excellent* | 739 (81.9) | 692 (93.6) | 47 (6.4) | 0.454 | 420 (60.8) | 271 (39.2) | **0.001** |
|  | *Very poor/*  *Poor/*  *Fair* | 163 (18.1) | 150 (92.0) | 13 (8.0) |  | 69 (45.4) | 83 (54.6) |  |
| **The pandemic is having a negative impact on your university career** | *Strongly disagree/*  *disagree* | 163 (18.1) | 153 (93.9) | 10 (6.1) | 0.483 | 100 (63.7) | 57 (36.3) | 0.267 |
|  | *Neutral* | 263 (29.2) | 249 (94.7) | 14 (5.3) |  | 139 (57.4) | 103 (42.6) |  |
|  | *Agree/*  *Strongly agree* | 476 (52.8) | 440 (92.4) | 36 (7.6) |  | 250 (56.3) | 194 (43.7) |  |
| **Family member who is an HCW** | *No* | 613 (68.0) | 578 (94.3) | 35 (5.7) | 0.098 | 333 (58.1) | 240 (41.9) | 0.926 |
|  | *Yes* | 289 (32.0) | 264 (91.3) | 25 (8.7) |  | 156 (57.8) | 114 (42.2) |  |
| **Belonging to a vulnerable group for COVID-19** | *No* | 868 (96.2) | 810 (93.3) | 58 (6.7) | 0.854 | 478 (58.9) | 333 (41.1) | **0.006** |
|  | *Yes* | 34 (3.8) | 32 (94.1) | 2 (5.9) |  | 11 (34.4) | 21 (65.6) |  |
| **A loved one belonging to a vulnerable group for COVID-19** | *No* | 323 (35.8) | 302 (93.5) | 21 (6.5) | 0.892 | 193 (63.9) | 109 (36.1) | **0.010** |
|  | *Yes* | 579 (64.2) | 540 (93.3) | 39 (6.7) |  | 296 (54.7) | 245 (45.3) |  |
| **Year of course** | *4^th^* | 200 (22.2%) | 192 (96.0%) | 8 (4.0%) | 0.359 | 104 (54.7) | 86 (45.3) | 0.615 |
|  | *5^th^* | 204 (22.6%) | 190 (93.1%) | 14 (6.9%) |  | 118 (61.5) | 74 (38.5) |  |
|  | *6^th^* | 260 (28.8%) | 239 (91.9%) | 21 (8.1%) |  | 141 (58.3) | 101 (41.7) |  |
|  | *Outside the prescribed times* | 238 (26.4%) | 221 (92.9%) | 17 (7.1%) |  | 126 (57.5) | 93 (42.5) |  |
| **Nationality** | *Italian* | 888 (98.4%) | 831 (93.6%) | 57 (6.4%) | **0.025** | 480 (57.9) | 349 (42.1) | 0.631 |
|  | *Other* | 14 (1.6%) | 11 (78.6%) | 3 (21.4%) |  | 9 (64.3) | 5 (35.7) |  |
| **Studying far from family home** | *No* | 385 (42.7%) | 360 (93.5%) | 25 (6.5%) | 0.663 | 216 (59.7) | 146 (40.3) | 0.057 |
|  | *Yes (same region)* | 303 (33.6%) | 280 (92.4%) | 23 (7.6%) |  | 172 (61.0) | 110 (39.0) |  |
|  | *Yes (different region)* | 214 (23.7%) | 202 (94.4%) | 12 (5.6%) |  | 101 (50.8) | 98 (49.2) |  |
| **Having job** | *No* | 813 (90.1%) | 758 (93.2%) | 55 (6.8%) | 0.680 | 437 (57.2) | 327 (42.8) | 0.139 |
|  | *Yes* | 89 (9.9%) | 84 (94.4%) | 5 (5.6%) |  | 52 (65.8) | 27 (34.2) |  |
| **Economic status** | *Good/excellent* | 819 (90.8%) | 768 (93.8%) | 51 (6.2%) | 0.108 | 449 (58.5) | 318 (41.5) | 0.320 |
|  | *Very poor/poor* | 83 (9.2%) | 74 (89.2%) | 9 (10.8%) |  | 40 (52.6) | 36 (47.4) |  |
| **Having a family member who is a healthcare worker in contact with COVID-19 patients** | *No* | 775 (85.9%) | 726 (93.7%) | 49 (6.3%) | 0.327 | 418 (57.7) | 306 (42.3) | 0.693 |
|  | *Yes* | 127 (14.1%) | 116 (91.3%) | 11 (8.7%) |  | 71 (59.7) | 48 (40.3) |  |
| **Pregnancy status** | *No* | 568 (99.6%) | 522 (91.9%) | 46 (8.1%) | **0.032** | 273 (52.0) | 252 (48.0) | 0.955 |
|  | *Yes* | 2 (0.4%) | 1 (50.0%) | 1 (50.0%) |  | 1 (50.0) | 1 (50.0) |  |

n=sample size

Figures are expressed as number (N) and percentages (%). Overall: column percentages. Descriptive analysis stratified by the outcomes: row percentages.

P-value obtained via Chi-squared test.

Abbreviations: HCW Healthcare worker

*expressed as median (interquartile range)

### Table S2. Descriptive analysis of items about flu vaccination, COVID-19 and COVID-19 vaccination and their relationships with the outcomes

| **Characteristic** |  | **Overall** | **Vaccine wicker** | | | **Extreme fear** | | |
| --- | --- | --- | --- | --- | --- | --- | --- | --- |
|  |  | **(n=902)** | **No**  **(n=842)** | **Yes**  **(n=60)** | **p** | **No (n=489)** | **Yes (n=354)** | **p** |
|  |  | N (%) | N (%) | N (%) |  | N (%) | N (%) |  |
| **Having received flu vaccination in the last three years** | *No* | 489 (54.2) | 454 (92.8) | 35 (7.2) | 0.492 | 264 (57.5) | 195 (42.5) | 0.928 |
|  | *At least once* | 255 (28.3) | 242 (94.9) | 13 (5.1) |  | 139 (58.2) | 100 (41.8) |  |
|  | *Each year* | 158 (17.5) | 146 (92.4) | 12 (7.6) |  | 86 (59.3) | 59 (40.7) |  |
| **Being aware of having received all the recommended paediatric vaccinations** | *No* | 21 (2.3) | 15 (71.4) | 6 (28.6) | **<0.001** | 9 (47.4) | 10 (52.6) | 0.349 |
|  | *Yes* | 878 (97.7) | 825 (94.0) | 53 (6.0) |  | 477 (58.1) | 344 (41.9) |  |
| **Having suffered from an adverse reaction after a vaccination** | *No* | 817 (90.6) | 770 (94.2) | 47 (5.8) | **0.001** | 447 (58.6) | 316 (41.4) | 0.294 |
|  | *Yes* | 85 (9.4) | 72 (84.7) | 13 (15.3) |  | 42 (52.5) | 38 (47.5) |  |
| **Having ever received the advice not to receive the flu vaccination** | *No* | 608 (67.4) | 574 (94.4) | 34 (5.6) | 0.066 | 332 (58.3) | 237 (41.7) | 0.773 |
|  | *Yes* | 294 (32.6) | 268 (91.2) | 26 (8.8) |  | 157 (57.3) | 117 (42.7) |  |
| **Having seen on social media the recommendation not to receive the flu vaccination** | *No* | 469 (52.0) | 431 (91.9) | 38 (8.1) | 0.069 | 255 (58.2) | 183 (41.8) | 0.897 |
|  | *Yes* | 433 (48.0) | 411 (94.9) | 22 (5.1) |  | 234 (57.8) | 171 (42.2) |  |
| **Fear of contracting flu personally: with regard to the participant own health** | *None/low* | 817 (90.6) | 764 (93.5) | 53 (6.5) | 0.538 | 467 (61.1) | 297 (38.9) | **<0.001** |
|  | *Moderate/high* | 85 (9.4) | 78 (91.8) | 7 (8.2) |  | 22 (27.8) | 57 (72.2) |  |
| **Fear of contracting flu personally: with regard to health of loved ones the participant is in contact with** | *None/low* | 281 (31.2) | 256 (91.1) | 25 (8.9) | 0.069 | 182 (70.0) | 78 (30.0) | **<0.001** |
|  | *Moderate/high* | 621 (68.8) | 586 (94.4) | 35 (5.6) |  | 307 (52.7) | 276 (47.3) |  |
| **Having ever received the advice not to receive the COVID-19 vaccination** | *No* | 426 (47.2) | 402 (94.4) | 24 (5.6) | 0.246 | 231 (58.3) | 165 (41.7) | 0.857 |
|  | *Yes* | 476 (52.8) | 440 (92.4) | 36 (7.6) |  | 258 (57.7) | 189 (42.3) |  |
| **Having ever received the advice not to receive the COVID-19 vaccination: by a relative** | *No* | 683 (75.7) | 644 (94.3) | 39 (5.7) | **0.045** | 362 (57.1) | 272 (42.9) | 0.351 |
|  | *Yes* | 219 (24.3) | 198 (90.4) | 21 (9.6) |  | 127 (60.8) | 82 (39.2) |  |
| **Having ever exhorted relatives/friends to follow preventive measures** | *No* | 104 (12.3) | 93 (89.4) | 11 (10.6) | **0.032** | 71 (68.3) | 33 (31.7) | **0.024** |
|  | *Yes* | 739 (87.7) | 700 (94.7) | 9 (5.3) |  | 418 (56.6) | 321 (43.4) |  |
| **Having been isolated for a suspected of confirmed case of COVID-19** | *No, never* | 590 (70.0) | 555 (94.1) | 35 (5.9) | 0.605 | 337 (57.1) | 253 (42.9) | 0.627 |
|  | *Yes, in the past* | 238 (28.2) | 223 (93.7) | 15 (6.3) |  | 144 (60.5) | 94 (39.5) |  |
|  | *Yes, currently* | 15 (1.8) | 15 (100.0) | 0 (0.0) |  | 8 (53.3) | 7 (46.7) |  |
| **Having been tested positive for COVID-19** | *No, never* | 776 (92.1) | 730 (94.1) | 46 (5.9) | 0.779 | 452 (58.2) | 324 (41.8) | 0.697 |
|  | *Yes, in the past* | 60 (7.1) | 56 (93.3) | 4 (6.7) |  | 34 (56.7) | 26 (43.3) |  |
|  | *Yes, currently* | 7 (0.8) | 7 (100.0) | 0 (0.0) |  | 3 (42.9) | 4 (57.1) |  |
| **A loved one tested positive for COVID-19** | *No, never* | 361 (42.8) | 342 (94.7) | 19 (5.3) | 0.730 | 218 (60.4) | 143 (39.6) | **0.013** |
|  | *Yes (not severely affected)* | 377 (44.7) | 352 (93.4) | 25 (6.6) |  | 224 (59.4) | 153 (40.6) |  |
|  | *Yes (severely affected)* | 105 (12.5) | 99 (94.3) | 6 (5.7) |  | 47 (44.8) | 58 (55.2) |  |
| **Fear of contracting COVID-19 personally: with regard to the participant own health** | *None/low* | 417 (49.5) | 392 (94.0) | 25 (6.0) | 0.938 | 308 (73.9) | 109 (26.1) | **<0.001** |
|  | *Moderate/high* | 426 (50.5) | 401 (94.1) | 25 (5.9) |  | 181 (42.5) | 245 (57.5) |  |
| **Fear of contracting COVID-19 personally: with regard to health of loved ones the participant is in contact with** | *None/low* | 26 (3.1) | 22 (84.6) | 4 (15.4) | 0.038 | 22 (84.6) | 4 (15.4) | **0.005** |
|  | *Moderate/high* | 817 (96.9) | 771 (94.4) | 46 (5.6) |  | 467 (57.2) | 350 (42.8) |  |
| **Having suffered from an adverse reaction after a flu vaccination** | *No* | 301 (72.9%) | 287 (95.3%) | 14 (4.7%) | 0.050 | 165 (58.5) | 117 (41.5) | 0.956 |
|  | *Yes* | 112 (27.1%) | 101 (90.2%) | 11 (9.8%) |  | 60 (58.8) | 42 (41.2) |  |
| **Kind of adverse reactions after flu vaccination#** | *Rhinitis* | 9 (8.0%) | 8 (88.9%) | 1 (11.1%) | 0.892 | 3 (42.9) | 4 (57.1) | 0.374 |
|  | *Fever* | 34 (30.4%) | 32 (94.1%) | 2 (5.9%) | 0.355 | 15 (51.7) | 14 (48.3) | 0.358 |
|  | *Cough* | 4 (3.6%) | 4 (100.0%) | 0 (0.0%) | 0.501 | 1 (33.3) | 2 (66.7) | 0.363 |
|  | *Muscular pain* | 35 (31.3%) | 34 (97.1%) | 1 (9.1%) | 0.095 | 20 (60.6) | 13 (39.4) | 0.800 |
|  | *Headache* | 31 (27.7%) | 30 (96.8%) | 1 (3.2%) | 0.147 | 18 (60.0) | 12 (40.0) | 0.876 |
|  | *Local reactions* | 67 (59.8%) | 58 (86.6%) | 9 (13.4%) | 0.117 | 41 (65.1) | 22 (34.9) | 0.103 |
|  | *Lymphadenopathy* | 7 (6.3%) | 7 (10.0%) | 0 (0.0%) | 0.367 | 1 (20.0) | 4 (80.0) | 0.070 |
| **Received flu vaccine in 2020-2021 season** | *No* | 343 (38.1%) | 328 (95.6%) | 15 (4.4%) | **0.031** | 181 (56.4) | 140 (43.6) | 0.455 |
|  | *Yes* | 558 (61.9%) | 513 (91.9%) | 45 (8.1%) |  | 308 (59.0) | 214 (41.0) |  |
| **Did the pandemic influence the decision to receive the flu vaccination?** | *No* | 655 (72.6%) | 613 (93.6%) | 42 (6.4%) | 0.699 | 364 (59.4) | 249 (40.6) | 0.329 |
|  | *Yes (negatively)* | 29 (3.2%) | 26 (89.7%) | 3 (10.3%) |  | 13 (48.1) | 14 (51.9) |  |
|  | *Yes (positively)* | 218 (24.2%) | 203 (93.1%) | 15 (6.9%) |  | 112 (55.2) | 91 (44.8) |  |
| **Having ever received the advice not to receive the flu vaccination from: #** | Relative | 148 (16.4%) | 133 (89.9%) | 15 (10.1%) | 0.063 | 78 (55.3) | 63 (44.7) | 0.479 |
|  | Friend | 70 (7.8%) | 62 (88.6%) | 8 (11.4%) | 0.095 | 38 (56.7) | 29 (43.3) | 0.823 |
|  | Acquaintance | 142 (15.7%) | 132 (93.0%) | 10 (7.0%) | 0.839 | 76 (56.7) | 58 (43.3) | 0.741 |
|  | General practitioner | 24 (2.7%) | 24 (100.0%) | 0 (0.0%) | 0.185 | 16 (76.2) | 5 (23.8) | 0.087 |
|  | Other physician | 25 (2.8%) | 22 (88.0%) | 3 (12.0%) | 0.276 | 9 (37.5) | 15 (62.5) | **0.039** |
| **Having ever received the advice not to receive the COVID-19 vaccination from: #** | Relative | 219 (24.3) | 198 (90.4) | 21 (9.6) | **0.045** | 127 (60.8) | 82 (39.2) | 0.351 |
|  | Friend | 174 (19.3%) | 159 (91.4%) | 15 (8.6%) | 0.246 | 99 (58.9) | 69 (41.1) | 0.787 |
|  | Acquaintance | 262 (29.0%) | 248 (94.7%) | 14 (5.3%) | 0.313 | 143 (57.2) | 107 (42.8) | 0.758 |
|  | General practitioner | 11 (1.2%) | 11 (100.0%) | 0 (0.0%) | 0.373 | 6 (66.7) | 3 (33.3) | 0.597 |
|  | Other physician | 33 (3.7%) | 27 (81.8%) | 6 (18.2%) | **0.007** | 21 (72.4) | 8 (27.6) | 0.110 |
| **Having seen on social media the recommendation not to receive the COVID-19 vaccination** | *No* | 240 (26.6%) | 214 (89.2%) | 26 (10.8%) | **0.002** | 132 (61.1) | 84 (38.9) | 0.284 |
|  | *Yes* | 662 (73.4%) | 628 (94.9%) | 34 (5.1%) |  | 357 (56.9) | 270 (43.1) |  |
| **Questions on stages of development of vaccines and clinical trial** | *At least one wrong* | 370 (43.9%) | 352 (95.1%) | 18 (4.9%) | 0.246 | 213 (57.6) | 157 (42.4) | 0.819 |
|  | *All right questions* | 473 (56.1%) | 441 (93.2%) | 32 (6.8%) |  | 276 (58.4) | 197 (41.6) |  |
| **Symptoms after COVID-19 infection** | *Asymptomatic* | 9 (13.4%) | 7 (77.8%) | 2 (22.2%) | 0.078 | 4 (44.4) | 5 (55.6) | 0.776 |
|  | *Mild* | 40 (59.7%) | 39 (97.5%) | 1 (2.5%) |  | 23 (57.5) | 17 (42.5) |  |
|  | *Moderate* | 18 (25.9%) | 17 (94.4%) | 1 (5.6%) |  | 10 (55.6) | 8 (44.4) |  |

n=sample size

Figures are expressed as number (N) and percentages (%). Overall: column percentages. Descriptive analysis stratified by the outcomes: row percentages. P-value obtained via Chi-squared test.

#possibility to select more options

### Table S3. Descriptive analysis of the items of the preventive score

| **Item** | **Overall** | **Vaccine hesitancy** | | | **Extreme fear** | | |
| --- | --- | --- | --- | --- | --- | --- | --- |
|  | **(n=902)** | **No**  **(n=842)** | **Yes**  **(n=60)** | **(n=902)** | **No**  **(n=842)** | **Yes**  **(n=60)** | **(n=902)** |
|  | **N (%)** | **N (%)** | **N (%)** | **N (%)** | **N (%)** | **N (%)** | **N (%)** |
| **Washing/sanitizing frequently their hands** |  |  |  |  |  |  |  |
| **October** |  |  |  |  |  |  |  |
| *No* | 23 (2.7%) | 23 (100.0%) | 0 (0.0%) | 0.222 | 20 (87.0) | 3 (13.0) | **0.004** |
| *Yes* | 820 (97.3%) | 770 (93.9%) | 50 (6.1%) |  | 469 (57.2) | 351 (42.8) |  |
| **August** |  |  |  |  |  |  |  |
| *No* | 97 (11.5%) | 92 (94.8%) | 5 (5.2%) | 0.731 | 63 (64.9) | 34 (35.1) | 0.141 |
| *Yes* | 746 (88.5%) | 701 (94.0%) | 45 (6.0%) |  | 426 (57.1) | 320 (42.9) |  |
| **Practising a proper respiratory etiquette** |  |  |  |  |  |  |  |
| **October** |  |  |  |  |  |  |  |
| *No* | 37 (4.4%) | 33 (89.2%) | 4 (10.8%) | 0.199 | 24 (64.9) | 13 (35.1) | 0.387 |
| *Yes* | 806 (95.6%) | 760 (94.3%) | 46 (5.7%) |  | 465 (57.7) | 341 (42.3) |  |
| **August** |  |  |  |  |  |  |  |
| *No* | 82 (9.7%) | 79 (96.3%) | 3 (3.7%) | 0.359 | 54 (65.9) | 28 (34.1) | 0.130 |
| *Yes* | 761 (90.3%) | 714 (93.8%) | 47 (6.2%) |  | 435 (57.2) | 326 (42.8) |  |
| **Using an app for contact tracing** |  |  |  |  |  |  |  |
| **October** |  |  |  |  |  |  |  |
| *No* | 382 (45.3%) | 353 (92.4%) | 29 (7.6%) | 0.063 | 213 (55.8) | 169 (44.2) | 0.229 |
| *Yes* | 461 (54.7%) | 440 (95.4%) | 21 (4.6%) |  | 276 (59.9) | 185 (40.1) |  |
| **August** |  |  |  |  |  |  |  |
| *No* | 453 (53.7%) | 417 (92.1%) | 36 (7.9%) | **0.008** | 254 (56.1) | 199 (43.9) | 0.220 |
| *Yes* | 390 (46.3%) | 376 (96.4%) | 14 (3.6%) |  | 235 (60.3) | 155 (39.7) |  |
| **Reducing the use of public transport** |  |  |  |  |  |  |  |
| **October** |  |  |  |  |  |  |  |
| *No* | 96 (11.4%) | 85 (88.5%) | 11 (11.5%) | **0.015** | 65 (67.7) | 31 (32.3) | **0.041** |
| *Yes* | 747 (88.6%) | 708 (94.8%) | 39 (5.2%) |  | 424 (56.8) | 323 (43.2) |  |
| **August** |  |  |  |  |  |  |  |
| *No* | 111 (13.2%) | 101 (91.0%) | 10 (9.0%) | 0.141 | 68 (61.3) | 43 (38.7) | 0.456 |
| *Yes* | 732 (86.8%) | 692 (94.5%) | 40 (5.5%) |  | 421 (57.5) | 311 (42.5) |  |
| **Increasing the cleaning of frequently touched surfaces** |  |  |  |  |  |  |  |
| **October** |  |  |  |  |  |  |  |
| *No* | 322 (38.2%) | 306 (95.0%) | 16 (5.0%) | 0.352 | 207 (64.3) | 115 (35.7) | **0.004** |
| *Yes* | 521 (61.8%) | 487 (93.5%) | 34 (6.5%) |  | 282 (54.1) | 239 (45.9) |  |
| **August** |  |  |  |  |  |  |  |
| *No* | 390 (46.3%) | 367 (94.1%) | 23 (5.9%) | 0.969 | 247 (63.3) | 143 (36.7) | **0.004** |
| *Yes* | 453 (53.7%) | 426 (94.0%) | 27 (6.0%) |  | 242 (53.4) | 211 (46.6) |  |
| **Meeting relatives/friends with masks and distancing** |  |  |  |  |  |  |  |
| **October** |  |  |  |  |  |  |  |
| *No* | 460 (54.6%) | 431 (93.7%) | 29 (6.3%) | 0.615 | 255 (55.4) | 205 (44.6) | 0.097 |
| *Yes* | 383 (45.4%) | 362 (94.5%) | 21 (5.5%) |  | 234 (61.1) | 149 (38.9) |  |
| **August** |  |  |  |  |  |  |  |
| *No* | 375 (44.5%) | 347 (92.5%) | 28 (7.5%) | 0.091 | 213 (56.8) | 162 (43.2) | 0.525 |
| *Yes* | 468 (55.5%) | 446 (95.3%) | 22 (4.7%) |  | 276 (59.0) | 192 (41.0) |  |
| **Often touching the face** |  |  |  |  |  |  |  |
| **October** |  |  |  |  |  |  |  |
| *No* | 633 (75.1%) | 597 (94.3%) | 36 (5.7%) | 0.603 | 365 (57.7) | 268 (42.3) | 0.724 |
| *Yes* | 210 (24.9%) | 196 (93.3%) | 14 (6.7%) |  | 124 (59.0) | 86 (41.0) |  |
| **August** |  |  |  |  |  |  |  |
| *No* | 583 (69.2%) | 549 (94.2%) | 34 (5.8%) | 0.855 | 334 (57.3) | 249 (42.7) | 0.528 |
| *Yes* | 260 (30.8%) | 244 (93.8%) | 16 (6.2%) |  | 155 (59.6) | 105 (40.4) |  |
| **Going to restaurants/bars or similar with people outside their household** |  |  |  |  |  |  |  |
| **October** |  |  |  |  |  |  |  |
| *No* | 507 (60.1%) | 485 (95.7%) | 22 (4.3%) | **0.016** | 289 (57.0) | 218 (43.0) | 0.468 |
| *Yes* | 336 (39.9%) | 308 (91.7%) | 28 (8.3%) |  | 200 (59.5) | 136 (40.5) |  |
| **August** |  |  |  |  |  |  |  |
| *No* | 200 (23.7%) | 184 (92.0%) | 16 (8.0%) | 0.156 | 109 (54.5) | 91 (45.5) | 0.250 |
| *Yes* | 643 (76.3%) | 609 (94.7%) | 34 (5.3%) |  | 380 (59.1) | 263 (40.9) |  |
| **Going to gatherings in indoor spaces** |  |  |  |  |  |  |  |
| **October** |  |  |  |  |  |  |  |
| *No* | 760 (90.2%) | 720 (94.7%) | 40 (5.3%) | **0.013** | 440 (57.9) | 320 (42.1) | 0.841 |
| *Yes* | 83 (9.8%) | 73 (88.0%) | 10 (12.0%) |  | 49 (59.0) | 34 (41.0) |  |
| **August** |  |  |  |  |  |  |  |
| *No* | 663 (78.6%) | 626 (94.4%) | 37 (5.6%) | 0.408 | 377 (56.9) | 286 (43.1) | 0.196 |
| *Yes* | 180 (21.4%) | 167 (92.8%) | 13 (7.2%) |  | 112 (62.2) | 68 (37.8) |  |

n=sample size

Figures are expressed as number (N) and percentages (%). Overall: column percentages. Descriptive analysis stratified by the outcomes: row percentages.

P-value obtained via Chi-squared test.

### Table S4. Statements about vaccinations in general and about COVID-19 and COVID-19 vaccinations and their relationships with vaccine hesitancy

| **Statement** |  | **Overall** | **Vaccine hesitancy** | | |
| --- | --- | --- | --- | --- | --- |
|  |  | **(n=902)** | **No**  **(n=842)** | **Yes**  **(n=60)** | **p** |
|  |  | N (%) | N (%) | N (%) |  |
| **Statement on vaccinations in general** | | | | | |
| **I do not have time to get vaccinated** | *Disagree* | 841 (93.2) | 784 (93.2) | 57 (6.8) | 0.351 |
|  | *Neutral* | 44 (4.9) | 43 (97.7) | 1 (2.3) |  |
|  | *Agree* | 17 (1.9) | 15 (88.2) | 2 (11.8) |  |
| **I am worried about the adverse effects of vaccinations** | *Disagree* | 803 (89.0) | 770 (95.9) | 33 (4.1) | **<0.001** |
|  | *Neutral* | 71 (7.9) | 56 (78.9) | 15 (21.1) |  |
|  | *Agree* | 28 (3.1) | 16 (57.1) | 12 (42.9) |  |
| **I think vaccinations are essential for my health** | *Disagree* | 9 (1.0) | 7 (77.8) | 2 (22.2) | **<0.001** |
|  | *Neutral* | 33 (3.7) | 26 (78.8) | 7 (21.2) |  |
|  | *Agree* | 860 (95.3) | 809 (94.1) | 51 (5.9) |  |
| **I do not think to be adequately informed about vaccinations** | *Disagree* | 734 (81.4) | 696 (94.8) | 38 (5.2) | **0.001** |
|  | *Neutral* | 96 (10.6) | 82 (85.4) | 14 (14.6) |  |
|  | *Agree* | 72 (8.0) | 64 (88.9) | 8 (11.1) |  |
| **I am afraid of needles** | *Disagree* | 824 (91.4) | 773 (93.8) | 51 (6.2) | **0.007** |
|  | *Neutral* | 42 (4.7) | 40 (95.2) | 2 (4.8) |  |
|  | *Agree* | 36 (4.0) | 29 (80.6) | 7 (19.4) |  |
| **Too many vaccinations can overload the immune system** | *Disagree* | 875 (97.0) | 818 (93.5) | 57 (6.5) | 0.433 |
|  | *Neutral* | 22 (2.4) | 20 (90.9) | 2 (9.1) |  |
|  | *Agree* | 5 (0.6) | 4 (80.0) | 1 (20.0) |  |
| **I do not think vaccines work** | *Disagree* | 896 (99.3%) | 838 (93.5%) | 58 (6.5%) | **0.011** |
|  | *Neutral* | 5 (0.6%) | 3 (60.0%) | 2 (40.0%) |  |
|  | *Agree* | 1 (0.1%) | 1 (100.0%) | 0 (0.0%) |  |
| **I think vaccines are dangerous** | *Disagree* | 896 (99.3%) | 840 (93.8% | 56 (6.3%) | **<0.001** |
|  | *Neutral* | 3 (0.3%) | 1 (33.3%) | 2 (66.7%) |  |
|  | *Agree* | 3 (0.3%) | 1 (33.3%) | 2 (66.7%) |  |
| **I do not trust who produces vaccines** | *Disagree* | 883 (97.9%) | 832 (94.2%) | 51 (5.8%) | **<0.001** |
|  | *Neutral* | 11 (1.2%) | 6 (54.5%) | 5 (45.5%) |  |
|  | *Agree* | 8 (0.9%) | 4 (50.0%) | 4 (50.0%) |  |
| **I do not trust who recommends vaccines** | *Disagree* | 896 (99.3%) | 839 (93.6%) | 57 (6.4%) | **<0.001** |
|  | *Neutral* | 4 (0.4%) | 2 (50.0%) | 2 (50.0%) |  |
|  | *Agree* | 2 (0.2%) | 1 (50.0%) | 1 (50.0%) |  |
| **I think that vaccines are essential for the health of the community** | *Disagree* | 13 (1.4%) | 10 (76.9%) | 3 (23.1%) | **<0.001** |
|  | *Neutral* | 3 (0.3%) | 0 (0.0%) | 3 (100.0%) |  |
|  | *Agree* | 885 (98.2%) | 831 (93.9%) | 54 (6.1%) |  |
| **Statement on COVID-19 and COVID-19 vaccinations** | | | | | |
| **It is essential that I get vaccinated to protect my family** | *Disagree* | 16 (1.8) | 6 (37.5) | 10 (62.5) | **<0.001** |
|  | *Neutral* | 22 (2.4) | 13 (59.1) | 9 (40.9) |  |
|  | *Agree* | 864 (95.8) | 823 (95.3) | 41 (4.7) |  |
| **It is essential that I get vaccinated to protect myself** | *Disagree* | 39 (4.3) | 26 (66.7) | 13 (33.3) | **<0.001** |
|  | *Neutral* | 88 (9.8) | 75 (85.2) | 13 (14.8) |  |
|  | *Agree* | 775 (85.9) | 741 (95.6) | 34 (4.4) |  |
| **It is essential that I get vaccinated to protect my community** | *Disagree* | 6 (0.7%) | 2 (33.3%) | 4 (66.7%) | **<0.001** |
|  | *Neutral* | 16 (1.8%) | 7 (43.8%) | 9 (56.3%) |  |
|  | *Agree* | 880 (97.6%) | 833 (94.7%) | 47 (5.3%) |  |
| **It is essential that I get vaccinated to protect patients** | *Disagree* | 4 (0.4%) | 2 (50.0%) | 2 (50.0%) | **<0.001** |
|  | *Neutral* | 11 (1.2%) | 3 (27.3%) | 8 (72.7%) |  |
|  | *Agree* | 887 (98.3 %) | 837 (94.4%) | 50 (5.6 %) |  |
| **I am worried to be infected with the SARS-CoV-2 through the vaccine** | *Disagree* | 881 (97.7%) | 827 (93.9%) | 54 (6.1%) | **<0.001** |
|  | *Neutral* | 15 (1.7%) | 10 (66.7%) | 5 (33.3%) |  |
|  | *Agree* | 6 (0.7%) | 5 (83.3%) | 1 (16.7%) |  |
| **I think the COVID-19 do not justify the need of a vaccine** | *Disagree* | 884 (98.0%) | 831 (94.0%) | 53 (6.0%) | **<0.001** |
|  | *Neutral* | 9 (1.0%) | 4 (44.4%) | 5 (55.6%) |  |
|  | *Agree* | 9 (1.0%) | 7 (77.8%) | 2 (22.2%) |  |
| **I think COVID-19 is not so severe as it is said** | *Disagree* | 880 (97.6%) | 827 (94.0%) | 53 (6.0%) | **<0.001** |
|  | *Neutral* | 16 (1.8%) | 12 (75.0%) | 4 (25.0%) |  |
|  | *Agree* | 6 (0.7%) | 3 (50.0%) | 3 (50.0%) |  |
| **I do not consider myself an important factor in the spread of the disease** | *Disagree* | 791 (87.7) | 746 (94.3) | 45 (5.7) | **0.006** |
|  | *Neutral* | 67 (7.4) | 59 (88.1) | 8 (11.9) |  |
|  | *Agree* | 44 (4.9) | 37 (84.1) | 7 (15.9) |  |
| **I do not think that my health is at risk in case of infection** | *Disagree* | 492 (54.6) | 458 (93.1) | 34 (6.9) | 0.162 |
|  | *Neutral* | 246 (27.3) | 235 (95.5) | 11 (4.5) |  |
|  | *Agree* | 163 (18.1) | 148 (90.8) | 15 (9.2) |  |
| **I do not think we will return to normal life until most people are vaccinated** | *Disagree* | 36 (4.0) | 29 (80.6) | 7 (19.4) | **<0.001** |
|  | *Neutral* | 71 (7.9) | 57 (80.3) | 14 (19.7) |  |
|  | *Agree* | 795 (88.1) | 756 (95.1) | 39 (4.9) |  |
| **I am worried about adverse effects of vaccinations** | *Disagree* | 746 (82.7) | 732 (98.1) | 14 (1.9) | **<0.001** |
|  | *Neutral* | 109 (12.1) | 86 (78.9) | 23 (21.1) |  |
|  | *Agree* | 47 (5.2) | 24 (51.1) | 23 (48.9) |  |
| **I do not think a safe vaccine can be developed in such a short time** | *Disagree* | 773 (85.7) | 758 (98.1) | 15 (1.9) | **<0.001** |
|  | *Neutral* | 91 (10.1) | 68 (74.7) | 23 (25.3) |  |
|  | *Agree* | 38 (4.2) | 16 (42.1) | 22 (57.9) |  |
| **I do not think an effective vaccine can be developed in such a short time** | *Disagree* | 779 (86.4) | 753 (96.7) | 26 (3.3) | **<0.001** |
|  | *Neutral* | 88 (9.8) | 70 (79.5) | 18 (20.5) |  |
|  | *Agree* | 35 (3.9) | 19 (54.3) | 16 (45.7) |  |
| **Currently, the knowledge of COVID-19 is insufficient for vaccine development** | *Disagree* | 836 (92.7) | 796 (95.2) | 40 (4.8) | **<0.001** |
|  | *Neutral* | 48 (5.3) | 31 (64.6) | 17 (35.4) |  |
|  | *Agree* | 18 (2.0) | 15 (83.3) | 3 (16.7) |  |
| **I am afraid I am allergic to some component of the vaccine** | *Disagree* | 806 (89.4) | 766 (95.0) | 40 (5.0) | **<0.001** |
|  | *Neutral* | 76 (8.4) | 60 (78.9) | 16 (21.1) |  |
|  | *Agree* | 20 (2.2) | 16 (80.0) | 4 (20.0) |  |
| **If the circulation of the virus decreased a lot, I would not get vaccinated** | *Disagree* | 844 (93.6) | 801 (94.6) | 43 (5.1) | **<0.001** |
|  | *Neutral* | 38 (4.2) | 29 (76.3) | 9 (23.7) |  |
|  | *Agree* | 20 (2.2) | 12 (60.0) | 8 (40.0) |  |
| **I would not want to be among the first individuals to get vaccinated** | *Disagree* | 741 (82.2) | 736 (99.3) | 5 (0.7) | **<0.001** |
|  | *Neutral* | 89 (9.9) | 71 (79.8) | 18 (20.2) |  |
|  | *Agree* | 72 (8.0) | 35 (48.6) | 37 (51.4) |  |
| **Vaccination should be mandatory** | *Disagree* | 102 (11.3) | 73 (71.6) | 29 (28.4) | **<0.001** |
|  | *Neutral* | 186 (20.6) | 172 (92.5) | 14 (7.5) |  |
|  | *Agree* | 614 (68.1) | 597 (97.2) | 17 (2.8) |  |
| **Getting vaccinated would make me feel safer around other people** | *Disagree* | 34 (3.8) | 20 (58.8) | 14 (41.2) | **<0.001** |
|  | *Neutral* | 77 (8.5) | 62 (80.5) | 15 (19.5) |  |
|  | *Agree* | 791 (87.7) | 760 (96.1) | 31 (3.9) |  |
| **Getting vaccinated is complicated for logistical reasons (times, places, commitments ...)** | *Disagree* | 715 (79.3) | 665 (93.0) | 50 (7.0) | 0.687 |
|  | *Neutral* | 122 (13.5) | 116 (95.1) | 6 (4.9) |  |
|  | *Agree* | 65 (7.2) | 61 (93.8) | 4 (6.2) |  |

n=sample size

Figures are expressed as number (N) and percentages (%). Overall: column percentages. Descriptive analysis stratified by vaccine hesitancy: row percentages.

Disagree=Strongly disagree/Disagree; Agree= Agree/Strongly agree.

P-value obtained via Chi-squared test.

### Table S5. Logistic regression models for vaccine hesitancy: univariable and multivariable regressions

|  | **Vaccine hesitancy** | | | |
| --- | --- | --- | --- | --- |
|  | **Univariable regressions** | | **Multivariable model** | |
|  | **OR (95% CI)** | **p-value** | **adjOR (95% CI)** | **p-value** |
| **Age** | 1.05 (0.97-1.14) | 0.233 | 1.11 (0.99-1.25) | 0.076 |
| **Female** | 2.18 (1.16-4.09) | **0.015** | 1.64 (0.79-3.42) | 0.183 |
| **Living alone** | 0.23 (0.03-1.72) | 0.153 | 0.15 (0.02-1.32) | 0.087 |
| **Worsening of economic status due to the pandemic** | 1.67 (0.91-3.08) | 0.100 | 2.06 (0.98-4.33) | 0.058 |
| **Having a family member who is a HCW** | 1.56 (0.92-2.67) | 0.100 | 1.93 (0.99-3.77) | 0.053 |
| **Survey before 21^st^ December** | 3.99 (2.34-6.80) | **<0.001** | 6.43 (3.26-12.67) | **<0.001** |
| **Being aware of having received all the recommended paediatric vaccinations** | 0.16 (0.06-0.43) | **<0.001** | 0.10 (0.03-0.39) | **0.001** |
| **Having suffered from an adverse reaction after a vaccination** | 2.96 (1.53-5.72) | **0.001** | 3.30 (1.43-7.64) | **0.005** |
| **Having seen on social media the recommendation not to receive the flu vaccination** | 0.61 (0.35-1.04) | 0.071 | 0.51 (0.26-0.997) | **0.049** |
| **Having ever received the advice not to receive the COVID-19 vaccination: by a relative** | 1.75 (1.01-3.05) | **0.047** | 2.40 (1.21-4.77) | **0.012** |
| **Fear of contracting COVID-19 personally: with regard to health of loved ones the participant is in contact with** | 0.33 (0.19-0.99) | **0.048** | 0.17 (0.05-0.58) | **0.005** |
| **Preventive score (October)** | 0.98 (0.96-0.99) | **0.008** | 0.98 (0.96-0.99) | **0.013** |

Abbreviations: adjOR adjusted Odds Ratio; CI Confidence Interval; HCW Healthcare worker; OR Odds Ratio

### Table S6. Logistic regression models for extreme fear of COVID-19: univariable and multivariable regressions

|  | **Extreme Fear** | | | |
| --- | --- | --- | --- | --- |
|  | **Univariable regressions** | | **Multivariable model** | |
|  | **OR (95% CI)** | **p-value** | **adjOR (95% CI)** | **p-value** |
| **Age** | 1.02 (0.97-1.08) | 0.413 | 1.01 (0.96-1.07) | 0.645 |
| **Female** | 1.95 (1.45-2.61) | **<0.001** | 1.85 (1.36-2.51) | **<0.001** |
| **Living with preschool children** | 0.45 (0.19-1.07) | 0.071 | 0.44 (0.18-1.08) | 0.073 |
| **Smoking** | 0.66 (0.44-0.98) | **0.037** | 0.68 (0.45-1.04) | 0.075 |
| **Very poor/poor/fair health status** | 1.86 (1.31-2.66) | **0.001** | 1.64 (1.12-2.39) | **0.011** |
| **Fear of contracting flu personally: with regard to the participant own health** | 4.07 (2.44-6.80) | **<0.001** | 3.06 (1.78-5.24) | **<0.001** |
| **Fear of contracting flu personally: with regard to health of loved ones the participant is in contact with** | 2.10 (1.54-2.86) | **<0.001** | 1.7 (1.22-2.37) | **0.002** |
| **A loved one tested positive for COVID-19: No, never** | Ref. |  | Ref. | 0.075 |
| **A loved one tested positive for COVID-19: Yes (not severely affected)** | 1.04 (0.78-1.40) | 0.788 | 1.03 (0.75-1.4) | 0.869 |
| **A loved one tested positive for COVID-19: Yes (severely affected)** | 1.88 (1.21-2.92) | **0.005** | 1.68 (1.06-2.67) | **0.029** |
| **Fear of contracting COVID-19 personally: with regard to health of loved ones the participant is in contact with** | 4.12 (1.41-12.07) | **0.01** | 2.6 (0.85-7.96) | 0.093 |

Abbreviations: adjOR adjusted Odds Ratio; CI Confidence Interval; OR Odds Ratio
